# Personal wellbeing among adolescents and youth in India

**DOI:** 10.3389/fpsyg.2022.914152

**Published:** 2022-08-25

**Authors:** Dhriti Ratra, Kamlesh Singh

**Affiliations:** ^1^Department of Psychiatry, University of Oxford, Oxford, United Kingdom; ^2^Department of Humanities and Social Sciences, Indian Institute of Technology Delhi, New Delhi, India

**Keywords:** Personal Wellbeing Index (PWI), adolescent wellbeing, youth wellbeing, India, socio-cultural experiences, happiness determinants

## Abstract

This study sought to explore the level of personal wellbeing and identified the determinants of happiness among Indian adolescents and youth. Data were collected from a sample of 495 participants (aged 11–23 years) residing in the National Capital Region of Delhi (Delhi-NCR), using the bilingual version (Hindi and English) of the Personal Wellbeing Index (PWI). Their PWI score was 80.06, indicating high happiness levels in the nonwestern normative PWI range. Domains of *personal relationship*s, *community connectedness*, and *safety* represented high overall wellbeing with the highest mean scores. Multivariate analysis showed that the least happy group on *life as a whole* domain was students aged 19–23 years as compared with the 11–14 and 15–18 years age group. Furthermore, men had higher happiness levels on *personal safety*, while women had higher scores on *life achievement*. The qualitative analysis illustrated the socio-cultural basis of these wellbeing determinants as rooted in the hierarchical social structures and collectivistic cultural orientation.

## Introduction

The World Health Organization classifies individuals aged between 10 and 24 years as “young people” [WHO Regional office for South-East Asia.(n.d.)]. This age group comprises early to late adolescence and youth which forms nearly 30.30% (~373 million) of India's total population (Office of the Registrar General Census Commissioner, India, 2011). Considering their significant contribution to the population of the country and its future economic growth, their health and wellbeing are of paramount importance to the various stakeholders including policymakers and educationists.

The transition from adolescence to adulthood marks an important period during which an individual develops essential behavior and thinking patterns, which could determine health-related outcomes (Lawrence et al., 2009). Several studies have shown that wellbeing-related behavioral patterns established during an individual's early adolescence continue through adulthood and later life impacting their physical and mental health (Currie et al., 2009; Patton et al., 2011). Studies have reported a positive association between high levels of satisfaction and happiness with global health, goal attainment, self-esteem, social identity, and community integration, which may act as stable characteristics continuing to early adulthood. Adolescents are especially prone to environmental influences (by peers, family, and society), which act as determining factors of their wellbeing - related behaviors (Sawyer et al., 2012). For example, a study by Suar et al. (2019) predicting factors of subjective wellbeing among Indian millennials found that satisfaction with personal relationships and emotional stability positively predicted their subjective wellbeing by providing a sense of calmness and fulfilled affiliation needs.

Among the various multifactor wellbeing models [e.g., psychological wellbeing, Ryff and Keyes (1995); PERMA model, Seligman (2012); mental health continuum, Keyes (2002), etc.], is the Personal Wellbeing (PWB) model, under the broad and multidimensional construct of Subjective Wellbeing (SWB). SWB explores people's interpretation of their own lives (their emotions and cognitive judgments), within which PWB specifically seeks to understand the overall life satisfaction of the individual in various domains of their life. It is measured using the psychometrically robust Personal Wellbeing Index (PWI), which assesses an individual's happiness in seven life domains, namely, standard of living, personal health, life achievement, personal relationships, personal safety, community connectedness, and future security, that act as first-level deconstructions of the global happiness measure: “life as a whole” (Lau et al., 2005).

Exploring an individual's wellbeing through the lens of socio-demographic and cultural determinants, the 2009/2010 HBSC WHO survey reported that the socio-demographic factors such as age and gender have been under-researched with regard to young people's wellbeing (Currie et al., 2009). A study by Singh et al. (2015a) found an age-related decline in levels of PWB among adolescents in India as they advanced from early to late adolescence. Similar results have been reported in Australian and Spanish adolescent samples (Tomyn et al., 2015; Tomyn and Cummins, 2011). However, the evidence on the association of age with various domains of personal wellbeing in India is limited. With regards to gender differences, different results have been found across various regions and cultures, for instance, Singh et al. (2015a) reported PWB to be higher in men than women, particularly in the PWI domains of personal safety and life achievement among the North Indian adolescent population. However, Daraei (2013) found no gender differences in the psychological wellbeing of young adults in a sample of Indian undergraduate students in Mysore city. Therefore, further research is needed to build more evidence and investigate the relation between socio-demographics and wellbeing.

Alongside socio-demographic factors, the cultural context of an individual also forms an integral part of their wellbeing (Diener et al., 2003). India is a highly collectivistic nation where most individuals define themselves through group goals, a “we” rather than “I” perspective. As collectivists, individuals in India value collaboration and constructive interdependence resulting in greater group harmony, security, and good social relationships, which form valued social constructs (Biswas-Diener et al., 2012). Research on happiness in collectivistic nations has shown that cultural norms predict the wellbeing of individuals. For example, a large study conducted by Suh et al. (1998) in 61 nations among more than 62,000 people showed that cultural norms strongly predicted life satisfaction among individuals in collectivistic nations. In another study, individuals with a collectivistic orientation show higher affective wellbeing at work (Rego and Cunha, 2009). In India, preliminary research has shown that sacrificing personal goals for group belongingness and other collectivistic themes such as relationship orientation and belief in the hierarchy may promote an individual's happiness (Biswas-Diener et al., 2012). It thus becomes imperative to study the trends and associations of wellbeing indicators in the specific cultural context of people in India.

The use of the native vernacular of a culture to understand the contextual nuances of a construct cannot be disputed. The issue of cultural meanings getting “lost in translation” has been depicted by some wellbeing scholars (e.g., Møller et al., 2015). For a detailed cultural understanding of the vernacular and socio-cultural nuances of wellbeing, qualitative research has proven to be a beneficial tool. For example, Møller et al. (2015) conducted focus group discussions in South Africa to investigate the meaning of the isiXhosa version of PWI depicting the wellbeing experiences of natives. Similar in-depth descriptive studies have been performed in other cultural contexts (e.g., Wiens et al., 2014; Thin, 2018).

In the Indian cultural context, a recent growing body of qualitative research has been conducted exploring the socio-cultural meaning of subjective wellbeing and happiness among children (Exenberger et al., 2019), among at-risk youth with traumatic life experiences (Exenberger and Reiber, 2020), and college students (Singh and Bandyopadhyay, 2022). However, qualitative research on the personal wellbeing indicators to understand the contextual nuances of wellbeing among adolescents and youth is still lacking in the Indian cultural context. Therefore, one of the main objectives of this study was to bridge this gap and examine through a qualitative study, the culture-specific trends in personal wellbeing among Indian adolescents and youth (aged 10–24 years as per the WHO classification).

Given this, this study employed a parallel mixed method design with the following aims:

(1) To quantitatively examine the level of personal wellbeing in a sample of Indian adolescents and youth and explore the demographic differences (age and gender-related) across the PWI domains.
(2) To develop a rich understanding of the facilitators and inhibitors of personal wellbeing among Indian adolescents and youth by studying their experiences using in-depth qualitative analysis.

## Methodology

This study employed a parallel mixed method research design with both quantitative and qualitative arms to holistically explore the research aims. The data collection methods and analysis approach are discussed in the “Procedure” section.

### Participants

A total of 541 participants took part in this study, from which we excluded 46 participants for submitting incomplete responses (missing data on at least one of the PWI domains). Our final sample comprised of 495 participants (*M*_age_ = 15.77 years, SD = 2.81).

As shown in Table 1, their age was collated into three age groups: 11–14 years, 15–18 years, and 19-23 years to look at the age-related developmental changes in young people. The majority of the participants in the sample belonged to the age groups of 15–18 years and 11–14 years, while 10 participants (2.02%) had missing data. There was a greater representation of school-going than college-going students in this study. Approximately, 59% of the participants were women, and the rest were men, while 17 (3.43%) had missing data on gender.

**Table 1:** Demographic sample characteristics.

**Demographic Characteristics**	**Participants *N* = 495**
	**Mean (SD)**
**Age in years (*****n*** **= 485)**	15.77 (2.81)
	***n*** **(%)**
11–14	191 (38.58)
15–18	217 (43.83)
19–23	37 (15.55)
**Sex (*****n*** **= 478)**	
Male	185 (37.40)
Female	293 (59.20)
**Level of Educational (*****n*** **= 495)**	
School students	405 (81.80)
College students	90 (18.20)

### The PWI measure

The Bilingual (English and Hindi) translated version of the PWI-School Children (PWI-SC) (Cummins and Lau, 2005) developed by Singh et al. (2015a) was utilized for this study. The measure assesses an individual's happiness in seven life domains, namely, standard of living, personal health, life achievement, personal relationships, personal safety, community connectedness, and future security that act as first-level deconstructions of the global happiness measure: “life as a whole” (Lau et al., 2005). The bilingual translated version was reported to be a reliable and valid instrument for measuring PWI among children and adolescents in India, yielding a Cronbach's α of 0.74, with a one-factor solution accounting for 41.29% of the variance and a moderate model fit. The scale items in the bilingual version were identical to the original scale and additionally provide space for qualitative responses after each question. Since a forced-choice question format restricts an in-depth exploration of the construct and limits the respondent to a set of options (e.g., rating 0–10), using an open-ended response format helped us capture the subjective perceptions of the respondents. Tomyn et al. (2013) studied the psychometric equivalence of the child and adult versions of the PWI, i.e., the PWI-SC and the PWI-A, and reported that they measure similar underlying constructs.

### Procedure

#### Data collection

Convenience sampling was used to collect the data from schools and colleges in the Delhi-NCR region of North India. Various education institutes were contacted to obtain permission from the principal/class teacher for data collection, and a consent letter was signed by the authorities. We took the informed consent of those aged 18 years and above, and the assent of the school students aged below 18 years. Participants were informed that their participation was voluntary, and they were free to withdraw from the research at any stage. They were also made aware that the results would be used only for research purposes, and confidentiality of data would be strictly maintained. The students were briefed about the study verbally, and a data collection booklet comprising the assent/consent form, a demographic information sheet, and the bilingual PWI form (each word/sentence written in Hindi and English languages) were distributed to them in the classroom. The participants were asked to respond to the PWI using the 0–10 scale and encouraged to elaborate on their responses. Therefore, both quantitative and qualitative data were obtained during data collection. The bilingual format is readily employed in India for all official government and private purposes, providing the participants with ease to write responses on the PWI scale in any language they were comfortable with. Any doubts that the participants had were clarified.

#### Quantitative data analysis

The overall PWI was reported as a mean score, calculated as per the guidelines of the International Wellbeing Group. Mean scores for the global “life as a whole” domain and the seven domains of PWI were also calculated. Ranks from 1 (highest happiness) to 7 (lowest happiness) were assigned to each of the PWI domains according to their mean scores to discern the low and high personal wellbeing domains. Furthermore, Multivariate Analysis of Variance (MANOVA) was conducted using SPSS version 20 to investigate the differential effect of demographic variables (age and gender) on the PWI domains.

#### Qualitative data analysis

Item-wise deductive content analysis of the data was conducted using the NVivo 12 Plus. The length of qualitative answers ranged from one sentence to five/ six sentences per domain. As an ongoing process, codes were generated and refined by both authors independently. Codes representing similar meanings were then categorized together and given suitable labels that captured the meaning appropriately, representing emergent themes. Authors actively deliberated the codes and refined them to ensure consistency. Any disagreements were resolved. The focus was on identifying specific inhibitors and facilitators of happiness as elaborated by the respondents. Like Singh et al. (2020), we categorized respondents' PWI scores as equal interval 11-point Likert scale responses (where higher scores reflect higher happiness). Categorizations of 0–4 (slightly happy), 5–6 (moderately happy), and 7–10 (highly happy) were formed wherein the lower score range (0–4) was indicative of inhibitors of happiness and the high score range (7–10) was indicative of the facilitators. The responses in the middle (5–6 score range) were a mix of the inhibiting and facilitating happiness responses.

## Results

Table 2 depicts the domain-wise PWI mean scores of the sample stratified by gender and age. The overall PWI of the sample was 80.06. The mean score on the global domain *happiness with life as a whole* was 79.30 (SD = 2.09), which was regarded as a separate item not included in the calculation of the overall PWI. Ranks from 1 (highest happiness) to 7 (lowest happiness) were assigned to each of the PWI domains according to their mean scores to discern the low and high personal wellbeing domains. Overall, happiness with *personal relationships* (1), *personal safety* (2), and *community connectedness* (3) were among the top-ranked items, whereas *future security* (7), *personal health* (6), and *standard of living* (5) received the lowest ranks. Table 2 further depicts demographically determined rank orders.

**Table 2:** Mean scores on the Personal Wellbeing Index (PWI) domains and their rank order.

**PWI Domains**	**Total Sample**		**Demographic Variables**									
			**Gender Distribution**				**Age-Wise Distribution**					
			**Males**		**Females**		**11–14 years**		**15–18 years**		**19–23 years**	
	**Mean (SD)**	**Rank Order**	**Mean (SD)**	**Rank Order**	**Mean (SD)**	**Rank Order**	**Mean (SD)**	**Rank Order**	**Mean (SD)**	**Rank Order**	**Mean (SD)**	**Rank Order**
Life as A Whole	79.30 (2.09)	-	77.50 (2.33)	-	80.90 (1.92)	-	79.90 (2.27)	-	82.10 (1.92)	-	70.40 (1.83)	-
1. Standard of Living	79.81 (1.89)	5	80.10 (1.82)	4	79.80 (1.95)	4	81.30 (1.89)	5	80.50 (1.86)	4	78.20 (1.66)	2
2. Personal Health	77.17 (2.25)	6	77.20 (2.40)	6	77.50 (2.16)	6	78.10 (2.20)	6	78.30 (2.34)	6	74.90 (1.97)	4
3. Life Achievement	81.00 (2.03)	4	79.30 (2.13)	5	82.80 (1.90)	2	83.40 (1.83)	2	83.90 (2.02)	2	67.10 (1.90)	7
4. Personal Relationships	84.52 (1.85)	1	84.30 (1.94)	1	85.00 (1.78)	1	86.20 (1.70)	1	86.70 (1.92)	1	77.40 (2.87)	3
5. Personal Safety	81.15 (2.19)	2	84.30 (2.06)	1	79.20 (2.29)	5	83.00 (2.20)	3	79.20 (2.44)	5	81.00 (1.72)	1
6. Community Connectedness	81.14 (2.23)	3	80.70 (2.35)	3	82.00 (2.15)	3	82.10 (2.14)	4	83.70 (2.29)	3	72.90 (2.14)	5
7. Future Security	75.4 (2.24)	7	74.00 (2.41)	7	76.50 (2.11)	7	76.60 (2.32)	7	76.20 (2.25)	7	72.20 (2.09)	6
Personal Wellbeing Index	80.06(0.29)	-	79.98(3.69)	-	80.40(3.02)	-	81.52 (3.26)	-	81.21 (3.68)	-	74.81 (4.59)	-

The range of mean values is from 0 to 100 with higher values depicting higher happiness levels.

Rank orders range from 1 (highest happiness) to 7 (lowest happiness) for all the PWI domains.

SD- Standard Deviations.

### Differential effects of demographic variables on the PWI domains

Multivariate analysis was conducted for all eight PWI domains to look at the differential effect of demographic variables (age and gender) on PWB. Seventeen participants did not report their gender, and ten participants did not report their age. These were excluded from the analysis totaling our sample to 474 participants.

Results showed statistically significant differences in wellbeing domains based on student's age [*F*(16, 928) = 3.90, *p* < 0.01, partial η^2^ = 0.06] and gender [*F*(8, 465) = 2.28, *p* < 0.01, partial η^2^ = 0.03]. Results on gender differences across the domains yielded statistically significant differences on life achievement [*F*(1, 472) = 3.93, *p* < 0.05, partial η^2^ = 0.008] with women possessing higher wellbeing than men; and on personal safety [*F*(1, 472) = 6.22, *p* < 0.05, partial η^2^ = 0.01] wherein men reported higher wellbeing.

Age-related differences were significant across the domains of life achievement [*F*(2, 471) = 19.60, *p* < 0.01, partial η^2^ = 0.07], personal relationships [*F*(2, 471) = 6.00, *p* < 0.01, partial η^2^= 0.25], community connectedness [*F*(2, 471) = 5.98, *p* < 0.01, partial η^2^ = 0.02], and life as a whole [*F*(2, 471) = 7.02, *p* < 0.01, partial η^2^= 0.02]. Multiple comparison analyses for age showed that the mean wellbeing scores were significantly different between age groups 11–14 and 19–23 years (*p* < 0.01) and between age groups 15–18 and 19–23 years (*p* < 0.01), but not statistically significant between the 11–14 and 15–18 age groups. Adolescents in the 15–18 age group scored highest on the wellbeing domains, whereas the young adults (19–23 age group) scored the lowest.

Examining the gender differences within each age group, it was found that in the 11–14 year group, gender differences were significant for the domains of life as a whole [*F*(1, 189) = 12.23, *p* < 0.01, partial η^2^= 0.06], community connectedness [*F*(1, 189) = 3.97, *p* < 0.05, partial η^2^= 0.02], and future security [*F*(1, 189) = 5.60, *p* < 0.05, partial η^2^= 0.03] with women reporting higher wellbeing in all domains than men. In the 15–18 age group, gender differences were significant for the domain of personal safety [*F*(1, 209) = 13.47, *p* < 0.01, partial η^2^= 0.06], where men scored higher wellbeing than women. In the 19–23 age group, gender differences were not statistically significant for any PWI domains.

### Qualitative analysis

The content analysis generated rich themes within each of the eight PWI domains. Table 3 depicts the response categorizations of happiness inhibitors (0–4 score range) and facilitators (7–10 score range,) depicting low and high levels of happiness, respectively. The responses in the middle (5–6 score range) denote a mix of the inhibiting and facilitating happiness responses.

**Table 3:** Content analysis depicting inhibitors and facilitators of happiness.

	**Inhibitors of Happiness (0–4 score range)**		**Facilitators of Happiness (7–10 score range)**	
	**Theme**	**References Number**	**Theme**	**References Number**
Life as a whole	Negative experiences with the family and community	26	Family belongingness	293
	Academic and work stress	21	Self-accomplishments	129
	Failure to accomplish aims	16	Community support	71
			Luxurious lifestyle	39
Standard of living	Monetary dependence on the family	90	Possession of assets and wealth	254
	Desire for possession of more assets	19	Non-monetary wealth	147
	Dearth of wealth	15	Satisfaction with basic life necessities	90
	Privilege comparison	6	Privilege comparison	30
Personal health	Frequency of illness and visits to the doctor	76	Self-care	134
	Physical appearances	28	Illness-wellness continuum	73
	Lack of self-care	27	Family care and support	41
	Ill health and a “diseased body”	20		
Achievement in life	Failure to accomplish aims	21	Optimistic self-beliefs	63
	Inhibiting self-virtues	19	Future responsibilities	39
	Uncertain future events	11	Support of significant others	24
			Societal expectations enhancing achievement	11
Personal relationships	Society's negative reactions	38	Belongingness with significant others	280
	Living far away from significant others	19	Self-personality factors defining relationships	68
	Self-personality inhibiting personal relations	19	Salient relationship virtues	56
	Academic and work pressure	11	Improving personal relations	15
Personal safety	Physical environment insecurity	134	Living in comfort zone	170
	Inhibiting gender societal norms	41	Protecting oneself	115
	Mishaps in media	14	Trust in government safety law enforcement	99
			Comparison with unsafe places	36
Part of community	Patriarchal community	34	Action-oriented outlook- changing the societal norms	227
	Dislike toward working-Importance of personal freedom	84	Belongingness with society	57
	Being away from home	87		
	Financial troubles	16		
Future security	Uncertainty regarding future	77	Ambitions in Life	193
	Negative life circumstances	13	Task-oriented outlook- initiatives for future happiness	141
	Professional competition with others	8	Self-capabilities to implement dreams	112
			Present as a testament to the future	49

#### Life as a whole

A majority of the participants gave high scores to this domain (7–10 range), indicating high levels of happiness. The major facilitators of happiness were family belongingness, community support, self-accomplishments, and a luxurious lifestyle. “*I am happy with my life in various areas. I have a good family, friends, studied in a good school & now in a good college. I feel satisfied with my life, although there are some things which give trouble but it is part of life”* (Female, Age-22, Score-8).

Friends and family acted as essential support networks motivating students toward achieving their goals. “*I read it myself at eight because I have very good friends who always help me whenever I am in trouble. They are very supportive. I live in hostel and miss my family very much. But they always call me and motivate me to do good things in my life”* (Female, Age-21, Score-8). Happiness with the community was expressed in the context of a positive learning environment at school- “*I am very happy because the life is very interesting in my school are so many activities in school have good teacher and good study”* (Male, Age-14, Score-9). Academic accomplishments and possession of life virtues (such as hard work and determination) emerged as strong facilitators of happiness, which were key to achieve success. “*I am quite happy and satisfied with my life as a whole. I want to achieve more for that I will do hard work so I am determined and confident”* (Male, Age-22, Score-9).

Only a few participants gave low scores (0-4 range) to this domain elucidating the inhibitors of happiness, namely, failure to accomplish aims, academic and work stress, and negative experiences with their family and community (such as gender discrimination). “*Happy with all things in life except the worry about the pressure to perform right now to have a successful future. The pressure to achieve goals is the reason of unhappiness or stress in life causing headaches*” (Male, Age: 20, Score: not reported).

Despite negative life circumstances, students reported an optimistic outlook where they chose to learn from their life experiences. “*Ups and downs are a part of life although I do feel sad when I can't sometimes get the things I enjoy. But I feel that if I have got this life, why not enjoy it fully.”* (Female, Age-16, Score-7).

#### Standard of living

Standard of living (SOL) is largely related to the possession of assets and wealth in the family. The majority of the participants who gave high scores to the domain typically belonged to families with a good socioeconomic status who could provide for them financially. “*I am very happy with annual income of my family I have nice DSLR NIKON D700 and also adidas shoes and branded t-shirts like Lee Cooper, Tommy Hilfiger, etc. I have more amazing things prove that I am happy”* (Male, Age-13, Score-9). While for others, happiness with basic life necessities acted as a facilitator of happiness in life- “*Whatever I could think of, necessities like comfort, education, family I have. I know what I can do with my life there's no regret for anything I do not dream of over comfort”* (Female, Age-20, Score-9).

Notably, 147 references emphasized that happiness arising from nonmonetary possessions such as spending quality time with significant others and life virtues is more important than monetary happiness (Table 3). Participants wrote- “*I am very happy with my standard of living because I have my parents love and trust which is much more important than any amount of money.”*(Female, Age-17, Score-10). “*Money is not important for happiness. We feel happy only when we achieve the things by hard-work. The most beautiful thing in my life is family.”* (Female, Age-17, Score-9).

Participants who gave a low score (0-4 range) mostly belonged to families who were financially not able to afford luxuries in life. The dearth of wealth, desire for more assets, and privilege comparison with others acted as primary inhibitors of happiness in their lives. “*I am not happy with the things I have because my family's financial situation isn't good. I see other people's things and imagine how it'd feel having them however my parents can't afford these things so I feel sad”* (Male, Age-16, Score-4).

#### Personal health

Synonymous with the other domains, a majority prescribed high scores (7-10 range) and elaborated on their active efforts toward the improvement of their health. Self-care in the form of good dietary choices, physical activity, and family support facilitated healthy living. “*I am very happy with my health because I eat my meals timely every day and try to include a variety of foods like pulses, grains, fruits for a well-balanced diet”* (Female, Age-16, Score-10); “*I am a sportsperson so I have to do everything to keep myself fit and healthy. My health is very good and I never encounter even fever”* (Male, Age-20, Score-10). Family support promoted healthy living and was linked to taking timely meals, avoiding unhealthy substances, and going for regular check-ups- “*My parents take care of my health. Being physically healthy makes me feel happy and reduces irritation in life”*(Female, Age-16, Score-8). Notably, throughout the themes, “health” was referred largely to physical fitness and not mental wellbeing. “*I am happy with my personal health because I have no ailments in my body. I just wish that in future too I remain disease-free.”* (Male, Age-14, Score-7).

In contrast, lack of self-care, frequently falling ill, and dissatisfaction with body appearances acted as inhibitors of happiness (0-4 range). Lack of self-care was related to unhealthy food choices, lack of physical exercise, and living in unhygienic environments. For example, “*Recently I have put on a lot of weight and completely stopped physical activity sedentary lifestyle due to work pressure, I want to rectify this”* (Age-21, Score-4). For participants with chronic ailments, the frequency of visits to the hospital determined their happiness with personal health.

Many students aspired to be healthier than they were now by improving self-care and changing their habits (such as being more athletic). “*If I start to care and take my health seriously then I think I would be able to give perfect 10*” (Male, Age-22, Score-7).

#### Achievement in life

Participants focused on academic pursuits, involvement in sports, and job opportunities when describing their life goals. Participants with a high score in this domain talked about their capabilities, support from significant others, societal expectations, and future responsibilities as essential motivators for goal attainment. Enumerating essential life skills, students wrote- “*It is quite impossible to be good at something you aspire to be good at. It always needs more practice and attention”* (Male, Age-21, Score-7). Furthermore, participants also reported feeling happy by remembering the praises they received from neighbors and family which acted as an essential motivator to achieve goals. Elucidating on family support, participants wrote-“*When I think about success, I feel very happy because I want to be an engineer and my parents always motivate and support me to achieve this dream”* (Male, Age-15, Score-9); “*I have full confidence in my capabilities and love and support of my parents through which I am able to work toward my goals”* (Male, Age-21, Score-8).

Unhappiness results from failure to accomplish aims, inhibiting self-virtues, and uncertainty regarding future events. Disappointed with failures, a participant wrote “*I am not so happy because I always try to improve myself but I do not get good results related to my study and I am worried about my parents what if I am not able to fulfill their dreams”* (Female, Age-15, Score-4). Anxiety and fear associated with future uncertainty were further impediments to happiness. “*I am happy about the dreams I have but the thought of whether I'll be able to fulfill my dreams makes me unhappy”* (Male, Age-17, Score-5). A few students also enumerated certain personality factors acting as an impediment to their success, for example, not working hard, procrastinating, and worrying about life events made them give low scores in the life achievement domain. “*I always escape from my responsibilities but always keep thinking of doing it but I am so lazy to do it”* (Male, Age-14, Score-4).

#### Personal relationships

Participants outlined their belongingness with significant others and essential virtues as factors facilitating happiness. Most of the responses focused on the interactions with family and friends acting as essential sources of happiness: “*I really like meeting the people I know in school and at home like my parents, siblings, friends, and relatives. These define personal relationship for me”* (Male, Age-18, Score-not reported); “*I have always valued my friendship is very highly. I didn't have many close friends in school but in college I became also very close circle and have had amazing experiences with them”* (Score-8). “Mutual trust,” “supportiveness,” “respect,” and “emotional bonding” were among the important virtues reported while describing these personal relationships. Personality factors such as being extrovert and optimistic were reported as harbingers of happiness in relationships.

In contrast, factors straining personal relationships were increasing academic and work stress, living away from home due to career demands, and society's negative reactions. Elucidating on the negative reactions hindering relationships, a 14-year-old reported: “*Most of the people in my life are selfish and create a lot of nuisance”* (Male, Score-4). Respondents also reported some personality factors which inhibited happiness. For example, being introvert and shy in expressing emotions emerged as a barrier to form close relationships (Male, Age-19, Score-4); “*When I am with people I tend to behave differently. I am not able to express myself.”* (Female, Score-5).

For a few, improving personal relationships contributed to happiness- “*Earlier I had many fights with my best friends, that was a lesson for me to not trust people blindly but now with the bunch of people I hang out now are really supportive and in need are always by my side”* (Female, Age: 14, Score: 10).

#### Personal safety

Participants reported feeling safest at home as families provided them with the protection they needed. For example- “*I am happy with my safety because my father drops me to school and takes more care of me.”* (Female, Age-14, Score-8). For others, trust in government safety laws made them feel physically secure when outside their homes. Describing it as “*achi kanoon vyavastha”* in Hindi (translated as “good law and order”), participants wrote- “*In today's society, we can get help from the police after registering a compliant. I am happy with the police protection around me.”* (Translated from Hindi; Male, Age-16, Score-10).

Advancing greater opportunities for women through changing patriarchal mindsets and a greater focus on women's safety made women happy- “*Very often I feel unsafe in my society. However, I am happy because the government has now strengthened the women safety laws”* (Female, Age-17, Score-5). Many talked about the necessity of self-protection through learning techniques of karate or even greater knowledge of the social behavioral norms to feel safer. For example, “*I follow the traffic rules, always walk on zebra crossings, do not touch the bare electricity wires to avoid getting electrocuted and because of these reasons I feel safe”* (Male, Age-16, Score-7).

Increasing crime rates, rape cases, discrimination against women, and incidents of bullying acted as inhibitors of safety, making participants give low scores on the domain. For example, “*I am unhappy about my safety because everyone takes advantage of me since I am a girl and boys on the street abuse and stare when I am walking. If I share it with my family, they won't allow me to go out of the house”* (Female, Age-15, Score-0). Due to such incidents, many women reported feeling afraid and on a constant lookout when outside their homes. Male participants also reported similar safety concerns- “*Living in a hostile city like Delhi, you don't feel particularly safe but I'm glad I am a boy as it is very difficult to grow as a girl in this male dominant society (I am not being sexist)”* (Age-14, Score-7). The need for stricter laws for female security and equality was highly recognized.

#### Part of community

Most participants understood this domain as synonymous with “personal relationships.” Belongingness with the family and nearness to home were the largely reported themes. Although the majority responded with a high score, it is possible that participants understood this domain as just “living far from home” and not connected with the community. Following responses demonstrate this issue- “*Since I live in Delhi and my home is also in Delhi I get to meet my family whenever possible. So it does not feel like doing something away from home”* (Male, Age-20, Score-6); “*I'm not happy about being away from home but I know that this is an opportunity that I need to make most of the college and education”* (Male, Age-21, Score-7).

A total of 227 references centered around changing the prevalent norms of society for the betterment of the community. By identifying specific problem areas such as “*widespread lack of efficiency of the government employees who do not work with competence thus disrupting the economy,”* participants described how they would work to disseminate knowledge and tackle societal issues, making them feel happy and a responsible part of their community. “*I feel very happy whenever I talk or play with underprivileged children they have so much talent. They're doing hard work”* (Female, Age-21, Score-9). Others demonstrated their belongingness with neighbors and friends as essential support networks during difficult life phases. According to a participant, working together in society makes it easier and faster to complete tasks such as keeping the environment clean (Male, Age-18, Score-10).

Patriarchy, financial troubles, and lack of self-confidence acted as barriers to working for the community. Many girls reported that they were not allowed to work outside the home and found themselves in a constant struggle to achieve their dreams as compared with their male counterparts- “*I would be very proud of myself if I go to work outside home. In this male dominant society, I would be able to find my place and help other girls in our society by motivating them to work outside. Working outside is not a sign of weakness rather makes us independent”* (Female, Age-16, Score-10).

#### Future security

Participants talked about personal ambitions, financial security, and their contributions to the country's growth and future. Personal ambitions centered around working for a cause and earning money which was seen as a pathway to a secure future “*I am happy with my future because I want to be engaged in social work for the community and become a respectable and successful person”* (Male, Age-17, Score-8). Possessing virtues of hard work, optimism, and confidence acted as essential facilitators of happiness with future security- “*Life gives a lot of good opportunities, I just have to grab onto them. After my experiences, I'm pretty confident I'll be able to create heaven for myself on earth”* (Score-9).

“*Planning”* and “*learning from one's experiences”* along with having the “*capability to implement one's dreams”* were often detailed as essential components toward a successful future. One participant mentioned conducting extensive research for her professional success (Age-20, Score-8), while others had the support of their parents and siblings. For some, their present situations acted as a testament to their future- “*I feel sad when I think about my future because we are taking water resource for granted which will make our future problematic and painful”*(Female, Age-17, Score-9). Others were hopeful about the future of the country's growth- “*I am very happy when I think about my future because our country is slowly progressing. In future, mud houses will transform into cemented ones, corruption will cease to exist and our future will be bright”* (Male, Age-16, Score-5).

Uncertainty about future events, insecure living conditions, and professional competition acted as stressors, which made participants prescribe low scores in this domain (8%). For example, “*I feel little afraid due to the raising competition in our country. I want to be a chartered accountant. I know that I have to work hard”* (Male, Age-13, Score-5); “*I am quite nervous for my future. I always get tensed whether I will achieve my long-term goal in future or not”* (Male, Age-13, Score-4).

## Discussion

This study aimed to examine the level of personal wellbeing among young people in India and their experiences using the PWI scale across seven life domains, namely, the standard of living, personal health, achievement in life, personal safety, personal relationships, community connectedness, and future security, that act as the first-level deconstruction of the global happiness measure: “life as a whole” (Lau et al., 2005).

As a global happiness measure of the PWI, the “life as a whole” domain is typically comprised of responses from all seven PWI domains. (Singh et al., 2015a) found that the domains of standard of living, community belongingness, personal health, and achievement in life predicted happiness with life as a whole among Indian adolescents, which were the top-ranked domains constituting the facilitators of happiness among young people in our sample. Similarly, other studies conducted with Chinese and Australian adolescent samples found that living standards, personal relations, and life achievement domains predicted happiness with life as a whole (Lau et al., 2005). Demographically, the age-related differences that we found were consistent with the Romanian population where Baltatescu and Cummins (2006) reported higher levels of PWI among high-school students than university students, demonstrating that the cross-cultural determinants overlap on the global PWI measure. A similar age-related decline over the lifespan has also been reported for subjective wellbeing in multiple studies and populations, e.g., Chinese (Xing and Huang, 2014) and British (Clark and Oswald, 2006) populations, demonstrating that age-related differences in wellbeing may show a similar picture across cultures. This could be true for gender differences in wellbeing as well, where similar to our findings, a survey in 60 nations found that younger women reported higher levels of overall life satisfaction than young men (Inglehart, 2002). Higher happiness with life as a whole has also been reported in female Spanish adolescent samples (Casas et al., 2012).

Interestingly, responses on nonmonetary happiness by young people in the sample depicting the importance of family belongingness were synonymous with the findings of another study in Chile, on family support, and PWB among school students. They found that intangible and social support from family was positively related to happiness in life (Schnettler et al., 2015). In a collectivistic culture such as India, the importance of family values, such as cooperation and harmony, and bonds are further exemplified by the preference of “We” over “I,” thus positively impacting wellbeing (Mosquera, 2015). Our participants' responses on improving personal relationships by learning from past mistakes provided insight into the strong meaning of relationships in a student's life, making the domain of family belongingness one of the best-understood items.

Another observation to note was that the participants largely used “community connectedness” interchangeably with “belongingness with the family.” Møller et al. (2015) conducted focus group discussions on PWI in South Africa where they also reported “community connectedness” as a problematic item, which resonated responses with the personal relationship domain. This discrepancy in the current sample may reflect conceptual issues with language where the questionnaire term “*doing things away from home”* might have been culturally misunderstood.

Responses on belongingness with the community highlighted the need to inculcate the value of social work among students. Government programs such as the “National Service Scheme (NSS)” instill the value of community work and collaboration and might be a step in this direction. In a similar vein, recent research on happiness in India has identified strategies to enhance the happiness levels of people, identifying a greater need for engagement of people in voluntary social services (Bhattacharyya et al., 2019).

Moving on, we found age-related differences in wellbeing. School-going adolescents (aged 11–18) ranked personal relationships and life achievement higher, while college-going students (aged 19–23) ranked personal safety and standard of living higher. This depicts that the differential social needs are based on the developmental challenges among these population cohorts.

Qualitative responses on the domain of personal safety seemed to be demographically unanimous. Although men had higher wellbeing than women on personal safety, their responses consisted of similar safety concerns as enlisted women. However, adolescent men reported their wellbeing to be higher on personal safety than women, which has been well documented in previous literature (e.g., Casas et al., 2012; May, 2001). This can be explained by the social environment of the participants in India. With 17,000 reported incidents of crime against women in Delhi, which is projected to reach 28,000 in 2020 (Dwivedi and Sachdeva, 2019), the city is surely not safe for women. Incidents of serious violence, physical abuse, and rape have been rampant with few internationally notorious gruesome rape cases. In such a situation, it is slightly satisfactory that the qualitative responses of women in the sample pointed to feel safe at their home, which is usually also not immune to such crimes.

The PWI score of this study (80.06) falls on the high end of the normative score range of both western and nonwestern samples representing high happiness levels among the young people of Delhi-NCR. Consistent with our findings, Singh et al. (2015a) reported high mean PWI scores (76.4-87.2) among 13-18-year-old Indian students. The western PWI normative score range is 70-80 points (e.g., The Australian Wellbeing Index Report, 2018, PWI: 75.1; International Wellbeing Group, 2013). It has been reported by researchers that the PWI score range of Asian countries is generally 10 points lower than western normative scores including studies from China (e.g., Chen and Davey, 2009, PWI: 64.4), Hong-Kong (Lau et al., 2005, PWI: 65.9), and Thailand (Yiengprugsawan et al., 2010, PWI: 75.7). These high scores reported by the study could perhaps be because the sample is representative of an urban setting (Delhi-NCR) where resources are more readily available than other areas in India affecting their wellbeing experiences. Consistent with the present research findings, another study from Delhi-NCR by (Singh et al., 2015b) upheld the view that slightly more Indians were flourishing and less languishing compared with the other groups such as the US and South African youths. Therefore, replicating our study in different parts of India would better ascertain the normative PWI score range among Indian adolescents and youth.

Comparison of wellbeing ratings with other nonwestern samples shows similar findings with regards to the domains of personal relationships and safety, which were among the highest ranked items and future security ranked among the lowest by many Asian cohorts including samples from Zhuhai, Hong Kong, and Macau (Chen and Davey, 2009). In their research on the optimal level of wellbeing, Oishi et al. (2009) explored the nuances of a high level of happiness (those in the top 10% of happiness scores on a wellbeing measure). Utilizing the World Values Survey Data, they found that a slightly lower level of happiness was associated with the most academic and financial success. A high level of happiness was associated with success in close personal relationships. This study lends support to this finding, wherein 79% of those who reported a score of “10” on the global PWI domain (169 participants) regarded personal relationships (family support and belongingness) as an essential facilitator of happiness.

Using the internationally recognized PWI tool, this study captured the wellbeing experiences among young people of Delhi-NCR. The analysis of the data was conducted robustly by researchers and discussed in detail so as not to let any personal biases confound this study. Using the survey as a methodology, a large geographical area was reached in the cosmopolitan capital city of Delhi-NCR, and holistic accounts of wellbeing were captured. The use of the psychometrically valid bilingual PWI scale made it possible for the participants to answer with ease enhancing their conceptual understanding of the questions.

We recognized a few limitations to the study as well. Although survey methodology is an effective tool to research a large geographical population, its effectiveness is limited by short responses. Interactive interview sessions or focus group discussions might have captured in-depth responses on wellbeing. Another shortcoming that limits the generalizability of the present results is the nature of the sample, as data were primarily collected from middle and upper-class residents of an urban North Indian city. Future studies can use face-to-face interviews and focus group discussions with a more diverse sample. Such studies may also qualitatively explore gender and age-related differences or similarities in the conceptualization of personal wellbeing and its determinants among Indians, thereby addressing another shortcoming of the present research. Another possibility for future research in the area can be to quantitatively explore the relationship between personal wellbeing and sociodemographic variables such as caste, socio-economic status, religion, stream or course of study, or type of school or college. These variables can affect the personal wellbeing of youth (e.g., Achour et al., 2015). Thus, the experiences of wellbeing among young people need to be documented from other subpopulations within India.

In brief, the take-home message from this research is that this study supports and validates the findings of existing research on the personal wellbeing of adolescents and youth in India. It depicts the importance of the cultural background of the individual when examining wellbeing in the Indian cultural context. There is a need to conduct research on personal wellbeing in different regions of India to holistically understand the culture-specific trends in personal wellbeing.

## Data availability statement

The raw data supporting the conclusions of this article will be made available by the authors, without undue reservation.

## Ethics statement

All procedures in this study thoroughly followed the ethical standards and guidelines of the institutional research committee (Indian Institute of Technology, Delhi), and the 1964 Declaration of Helsinki ethical guidelines. Voluntary written informed consent to participate in the study was obtained from all participants 18 years and above, assent was obtained from minors, and formal permission was taken from the school administration. The data was collected ensuring ethical guidelines. All participants were made aware that the results would be used only for research purposes and confidentiality of data would be strictly maintained.

## Author contributions

KS conceptualized the study, collected the data, and oversaw the direction of the research. DR designed the research methodology, conducted the quantitative and qualitative analysis of the data, interpreted the results, and took the lead in writing the manuscript. KS and DR were involved in the qualitative content analysis, collaborated, and provided critical feedback to help shape the final version of the manuscript. Both authors contributed to the article and approved the submitted version.

## Conflict of interest

The authors declare that the research was conducted in the absence of any commercial or financial relationships that could be construed as a potential conflict of interest.

## Publisher's note

All claims expressed in this article are solely those of the authors and do not necessarily represent those of their affiliated organizations, or those of the publisher, the editors and the reviewers. Any product that may be evaluated in this article, or claim that may be made by its manufacturer, is not guaranteed or endorsed by the publisher.
